# Pseudotemporal ordering of spatial lymphoid tissue microenvironment profiles trails Unclassified DLBCL at the periphery of the follicle

**DOI:** 10.3389/fimmu.2023.1207959

**Published:** 2023-08-23

**Authors:** Claudio Tripodo, Giorgio Bertolazzi, Valeria Cancila, Gaia Morello, Emilio Iannitto

**Affiliations:** ^1^ Tumor Immunology Unit, Department of Sciences for Health Promotion and Mother-Child Care "G. D'Alessandro", University of Palermo, Palermo, Italy; ^2^ Histopathology Unit, Institute of Molecular Oncology Foundation (IFOM) ETS - The AIRC Institute of Molecular Oncology, Milan, Italy; ^3^ Department of Economics, Business, and Statistics, University of Palermo, Palermo, Italy; ^4^ Department of Oncology, Hematology and Bone Marrow Transplants Unit La Maddalena, Palermo, Italy

**Keywords:** digital spatial profiling, lymphoid tissue microenvironment, pseudotemporal ordering, diffuse large B cell lymphoma, cell-of-origin

## Abstract

We have established a pseudotemporal ordering for the transcriptional signatures of distinct microregions within reactive lymphoid tissues, namely germinal center dark zones (DZ), germinal center light zones (LZ), and peri-follicular areas (Peri). By utilizing this pseudotime trajectory derived from the functional microenvironments of DZ, LZ, and Peri, we have ordered the transcriptomes of Diffuse Large B-cell Lymphoma cases. The apex of the resulting pseudotemporal trajectory, which is characterized by enrichment of molecular programs fronted by TNFR signaling and inhibitory immune checkpoint overexpression, intercepts a discrete peri-follicular biology. This observation is associated with DLBCL cases that are enriched in the Unclassified/type-3 COO category, raising questions about the potential extra-GC microenvironment imprint of this peculiar group of cases. This report offers a thought-provoking perspective on the relationship between transcriptional profiling of functional lymphoid tissue microenvironments and the evolving concept of the cell of origin in Diffuse Large B-cell Lymphomas.

## Introduction

Diffuse large B-cell lymphomas (DLBCL) are phenotypically and genetically heterogeneous.

Applying a cell-of-origin (COO) classification algorithm to the gene expression profile of DLBCLs segregates the cases into two major subgroups (1). Germinal Center B Cell-like (GCB) DLBCL show a high level of expression of genes characteristic of physiological GC reaction molecular programs while Activated B Cell-like (ABC) DLBCL express genes typical of mitogenically-activated B-cells, are enriched in plasma cell-related programs, and regarded as embodying post-GC dynamics. A third subgroup of DLBCL is left out from the GCB/ABC dichotomy, which does not express either set of genes at a high level and is accordingly named Unclassified or Type-3 (2). Based on the transcriptional profile similarity, GCB and ABC DLBCL are considered frozen in a different stage of the functional modulation pathway engaging B-cells in their GC journey towards plasma cells or memory B (3). At difference, the Unclassified/Type-3 DLBCL group has been considered as possibly consisting of more than one type of DLBCL and populated mainly by borderline cases not assigned by the clustering algorithm, yet actually belonging to the ABC or GCB group (2). Several subsequent studies, applying different profiling platforms and classification algorithms, reproduced this molecular DLBCL tripartition, with the GCB representing the largest group (46-58%), followed by ABC (27-40%) and Unclassified/Type-3 (10-22%) (4–7). Retrospective studies reported that GCB DLBCL show a significantly better overall survival (OS) than ABC; conversely, the Unclassified/Type-3 group outcome varies widely among different retrospective series, showing an OS curve comparable to that of ABC or GCB DLBCL or lying in between (4–7). Of note, in two large prospective series, each including over a thousand patients, the Unclassified/Type-3 DLBCL showed an outcome comparable to that of the ABC group (8, 9). Recently, comprehensive genomic analysis with different platforms led to a genetic subtype classification of DLBCLs (6, 10, 11). With the caveat that nearly half of the cases remained unclassified (6, 12), the genetic classification highlighted that each of the three DLBCL COO subgroups included multiple genetic profiles. In particular, the Unclassified/Type-3 COO came out to be enriched for the BN2 genetic subtype that accounted for over one-third of cases, and comprised also cases belonging to ST2, EZB, MCD, A53 genetic clusters (12). BN2 DLBCL are characterized by mutations that activate NOTCH2 or inactivate the NOTCH antagonist SPEN, frequently co-occurring with BCL6 translocations. Genetic aberrations targeting regulators of the NF-κB pathway are another prominent feature of BN2 DLBCL. Mutations targeting components of the BCR-dependent NF-kB pathway (PRKCB, BCL10, TNFAIP3, TNIP1) occur in over 50% of cases predicting that these tumours rely on B-cell receptor-dependent NF-κB activation and could be vulnerable to antagonists of B-cell receptor signalling (6). The genomic profile of the BN2 cluster closely reminds that of Marginal Zone Lymphoma (MZL) and transformed MZL. All BN2 cases were confirmed to display a canonical DLBCL histological picture, suggesting that the Unclassified/Type-3 COO, besides misclassified ABC and GCB cases, may host a distinct subset of DLBCL (6). However, whether the Unclassified/Type-3 group is merely the wastebasket of gene expression profile (GEP) classification algorithms or the profile of DLBCL originating from discrete functional differentiation stages and microenvironmental settings has not been elucidated.

We have exploited the spatial transcriptional profiling (1824 genes, cancer transcriptome atlas panel, https://www.nanostring.com/products/geomx-digital-spatial-profiler/geomx-rna-assays/geomx-cancer-transcriptome-atlas/) of 15 microregions (Regions of Interest, ROIs) relative to GC dark zone (DZ, n=5) and light zone (LZ, n=5) microenvironment and peri-follicular (Peri n=5) areas. We used a pseudotime algorithm called PhenoPath (13) to learn about the biological progression that characterizes the regions of interest. In the context of single-cell analysis, pseudo time is a computational construct that is used to order cells along a temporal trajectory, based on the similarity of their gene expression profiles. Pseudo time analysis aims to capture the temporal ordering of cells based on the expression changes of key genes, without directly measuring the time point at which the cells were collected. Pseudo time analysis can help to reveal the cellular processes that are active during different stages of development or differentiation, and can provide insights into the regulatory networks that govern these processes. The recent development of a pseudotime algorithm calibrated for bulk RNA-seq data (13) allowed us to extract the latent temporal information from the spatial profiling of the ROIs.

## Results and discussion

**Figure 1:**
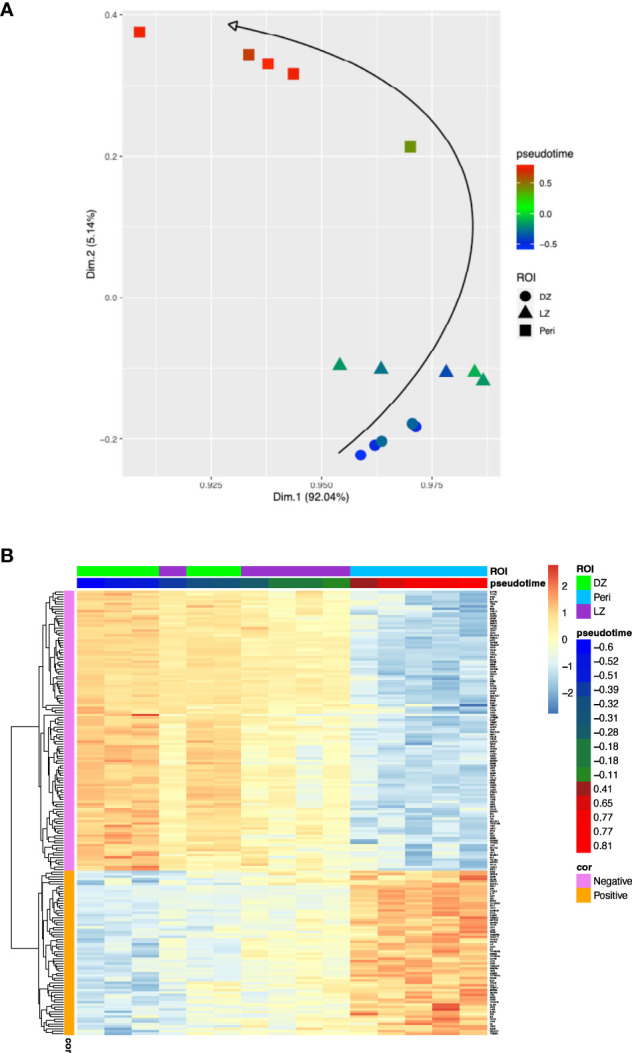
**(A)**, Two-dimensional principal component projection produces a trajectory among DZ, LZ, and Peri ROIs. The color gradient of points reflects the pseudotime estimated values. **(B)**, Expression heatmap of the 184 genes significantly correlated with the pseudotime over 15 ROIs. The 15 ROIs (columns) are ordered according to the pseudotime estimation.

**Figure 2:**
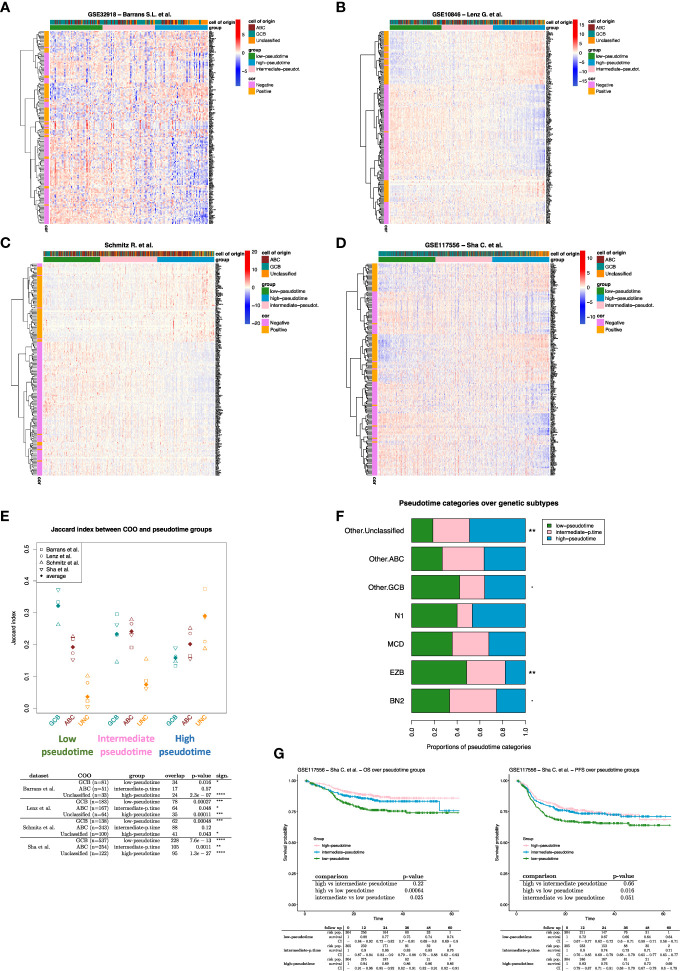
**(A-D)** Expression heatmap of the 184 pseudotime-related genes. The DLBCL cases (columns) are ordered according to their pseudotime-related score (see methods). Three pseudotime groups have been identified by applying the tertile separation on pseudotime-related scores (i.e., low, intermediate, and high pseudotime groups). **(E)** Jaccard similarity index between COO and pseudotime groups over DLBCL datasets. Unclassified cases strongly enrich all the high-pseudotime groups. While GCB cases enrich all the low-pseudotime groups (Fisher p-values are shown in the table). **(F)** Proportions of pseudotime categories over genetic subtype groups. The Fisher exact test has been applied to evaluate the association between genetic subtypes and pseudotime categories (**Supplementary Table 4**). “Other.ABC,” “Other.GCB,” and “Other.Unclassified” are cases that lack genetic subtype classification and only have COO classification. **(G)** Survival analysis on DLBCL cases from Sha et al. dataset. Patients were divided into three groups according to the tertiles of their pseudotime-related scores (i.e., low, intermediate, and high pseudotime groups). if p-value < 0.1 *; if p-value < 0.05; ** if p-value < 0.01; *** if p-value < 0.001; **** if p-value < 0.0001.

Using PhenoPath we estimated the pseudotemporal values from the bulk gene-expression matrix (**Supplementary Table 1**). Based on the pseudotime estimation (see methods), we obtained a pseudotemporal ordering of the individual ROIs. The pseudotime trajectory extended from DZ towards Peri regions, showing a clear association between pseudotemporal order and the spatial and functional ROI features (**Figure 1A**). Pseudotemporal ordering of digital spatial profiling ROIs enabled the identification of a pseudotime-associated gene signature composed of 184 genes significantly correlated with pseudotime (68 positively and 116 negatively) (**Figure 1B**; **Supplementary Table 1**). These 184 genes are representative of discrete variations in the ROI transcriptome along the calculated pseudotime ordering, reflecting variations in the underlying biology of the profiled microregions. The genes positively associated with pseudotime were mostly enriched in TNF signaling pathway genes (i.e. *NFRSF14, TNFRSF25, TNFRSF1A, TNFRSF1B*) and in genes involved in the negative regulation of T-cell activation (i.e. *CD274, VSIR, LAG3, IDO1* – **Supplementary Table 2**) while those inversely associated with pseudotime were enriched in cell proliferation, DNA damage repair, and B-cell receptor signaling programs (**Supplementary Tables 2, 3**). In the attempt to investigate the effects of the pseudotime trajectory derived from the transcriptional profiling of functional microenvironments of a reactive lymphoid tissue on the ordering of DLBCL transcriptomes, we applied the 184-genes pseudotime signature to four independent gene expression-profiled DLBCL cohorts (4–7) (**Figures 2A–D**). The four cohorts characterized by different case selection criteria and gene expression profiling technologies (Illumina, Affymetrix), displayed a generally conserved significance of the GCB vs ABC comparison in terms of OS (**Supplementary Figures 1A–D**). At odds, the fractions and prognostic behavior of Unclassified/Type-3 COO clusters in the four series were quite different (**Supplementary Figures 1A–D**), suggesting that this group of DLBCL might encompass a remarkable biological heterogeneity. By applying the pseudotime-associated microenvironment signature according to the tertile distribution of the cumulative expression of genes negatively and positively associated with pseudotime, DLBCL cases of the four cohorts displayed a common trend towards the enrichment of GCB cases in the *low-pseudotime* tertile (**Figure 2E**) a rather heterogeneous distribution of ABC cases, and a clear enrichment of Unclassified/Type-3 COO cases in the *high-pseudotime* tertile (**Figure 2E**). Consistently, the distribution of DLBCL genetic subgroups according to Schmitz across the low-, intermediate-, and high-pseudotime tertiles showed significant enrichment of EZB and other GCB genetics in the low-pseudotime group (**Figure 2F**; **Supplementary Table 4**). At the same time, BN2 was slightly enriched in the intermediate-pseudotime group, and other Unclassified genetics were detected across the three pseudotime categories, further indicating their mirroring of divergent biologies. We further explored if the pseudotime tertile hierarchy could rank DLBCL in groups with different prognosis. This hypothesis was tested in the series by Sha et al. (GSE117556) (7), which offers distinctive features that are relevant to this extent: 1) a large prospective clinical data-set of 928 18-years or older DLBCL patients, with a centralized gene expression profiling and pathological review, eligible for anthracycline-based treatment; 2) 30 months Progression-Free Survival (PFS) in line with the best results of the recent phase 3 trials on DLBCL; 3) COO classification refined retrospectively with the same method, taking advantage of higher quality samples and improved data normalization over the complete data-set (7). Most importantly, in this large series, the COO classification failed to identify groups with significantly different prognosis (**Supplementary Figures 1E, F**). The application of trichotomization of the series according to pseudotemporal scoring revealed significant prognostic differences between pseudotime-low and -high tertiles, with cases displaying high pseudotime ordering faring significantly better in terms of OS and PFS (**Figure 2G**; **Supplementary Table 5**). The different prognostic performance of COO and pseudotemporal ordering in this setting suggests that the transcriptional modulations represented in the spatial profiling of diverse GC and extra-follicular microregions may adequately cope with the wide continuum of DLBCL, at least in series including cases with DZ-related transcriptional profiles (i.e. molecular high grade).

In this study, a potential bias inherent with the small sample size of spatially profiled microregions should be considered as a note of caution. Nonetheless, the results indicate that the apex of the pseudotemporal trajectory resulting from spatial profiling intercepts a discrete peri-follicular biology characterized by the enrichment of molecular programs fronted by TNFR signaling and inhibitory immune checkpoint overexpression and corresponding to DLBCL cases enriched in the Unclassified/type-3 COO category, opening an issue regarding the potential extra-GC imprint of this heterogeneous group. An accurate biomolecular characterization of this hitherto neglected subset of DLBCL might pave the way for deciphering their biological and prognostic determinants.

## Materials and methods

### Digital spatial profiling

As described in our previous work (14), the transcriptional landscape of 15 different spatially-resolved regions of interest (ROIs) of the tonsil (5 peri/inter-follicular ROIs, 5 DZ, and 5 LZ ROIs from morphologically normal follicles) was determined by Digital Spatial Profiling on slides stained with CD271/NGFR (as an follicular dendritic cells marker to highlight the LZ) and CD20 (as a B-cell marker). The 15 selected and segmented ROIs were profiled using a GeoMx Digital Spatial Profiler (DSP) (NanoString, Seattle, WA) applying the Cancer Transcriptome Atlas panel (https://www.nanostring.com/products/geomx-digital-spatial-profiler/geomx-rna-assays/geomx-cancer-transcriptome-atlas/).

### Statistical analysis

Raw counts were normalized against the 75th percentile of signal from their own ROI. The R package Phenopath (15) has been used to estimate pseudotime values from bulk gene expression data as described by Campbell and Yau (13). We choose of the Phenopath algorithm for the pseudotime estimation because the standard pseudotime algorithms require single-cell data as input, while Phenopath can be used also on bulk RNA-seq data. Therefore, we consider a pseudotemporal ranking of ROIs based on pseudotime estimations. The temporal trajectory has been highlighted on a PCA projection performed on normalized data using the FactoMine R package. The Spearman correlation coefficients have been calculated between gene expression and pseudotime estimated values. The Bonferroni correction for multiple comparisons has been applied to evaluate the p-value significance of correlation coefficients (FWER controlled at 5% level). The pseudotime significantly correlated genes compose the pseudotemporal signature (**Supplementary Table 1**).

The pseudotemporal signature was assessed in the following DLBCL datasets: Barrans et al. (GSE32918) (4), Lenz et al. (GSE10846) (5), Schmitz et al. (6), and Sha et al. (GSE117556) (7). The Barrans and the Sha datasets have been downloaded from GEO using the GEOquery R package. Regarding the datasets of Barrans, Sha, and Schmitz, we maintained the normalization proposed by the authors. The Lenz et al. (GSE10846) expression matrix has been obtained from the CEL file available on GEO and it has been normalized using the gcrma package.

To order DLBCL patients according to the pseudotemporal gene signature, we have calculated a pseudotime-related score that combines the expression of the pseudotime-signature genes with the correlation coefficients previously calculated on the DSP dataset. Considering the patient-*j*, his pseudotime-related score is calculated as:


$${\text{score}}_{j}=\sum_{i=1}^{n}{\hat{\rho }}_{i}\cdot x_{i}$$

Where ρ_i_ is the Spearman correlation coefficient between the expression of gene-*i* and pseudotime values (it has been previously calculated on the DSP dataset), *x_i_* is the expression of gene-*i* in the DLBCL dataset, and *n* is the number of genes of the pseudotemporal signature. The coefficients ρ_i_ allow us to weight the gene expression considering how strong is the correlation between each gene and the pseudotime values. Using the score, each DLBCL cohort was divided into low-pseudotime, intermediate-pseudotime, and high-pseudotime.

The Jaccard similarity index has been calculated to measure the association between the cell of origin (COO) and the pseudotime groups in DLBCL datasets. The Fisher exact test has been used to evaluate the association between pseudotime groups and COO over DLBCLs, and the association between pseudotime groups and genetic subtypes in the Schmitz dataset.

The prognostic power of the pseudotemporal signature has been tested on the Sha et al. dataset. The overall Survival (OS) and the progression-free survival (PFS) have been compared among the three pseudotime groups. Kaplan-Meier method has been used to estimate the survival functions among groups, and the log-rank test has been used to test the differences in the overall survival between the identified groups. Before calculating the log-rank test, the cox-pzh test was used to test the proportional hazard assumption (**Supplementary Table 5**). We have adapted a multivariate Cox model including the pseudotime groups, the COO classes, and the IPI-risk classes (i.e., low, medium, and high risk) to verify that the pseudotime group variable maintains its significance. The whole survival analysis has been carried out through the survival R package. All statistical analyses were performed using R software (v 4.0.2) (http://www.R-project.org).

## Data availability statement

The original contributions presented in the study are included in the article/**Supplementary Material**. Further inquiries can be directed to the corresponding author.

## Ethics statement

Ethical approval was not required for the study involving humans in accordance with the local legislation and institutional requirements. Written informed consent to participate in this study was not required from the participants or the participants’ legal guardians/next of kin in accordance with the national legislation and the institutional requirements.

## Author contributions

CT, GB, EI conceptualization, data analysis, writing; VC, GM data analysis. All authors contributed to the article and approved the submitted version.
