# Supplementary figures and images for: Pseudotemporal ordering of spatial lymphoid tissue microenvironment profiles trails Unclassified DLBCL at the periphery of the follicle
Source: Front Immunol. 2023 Aug 23;14:1207959. doi: 10.3389/fimmu.2023.1207959 (PMC10482233; doi:10.3389/fimmu.2023.1207959)

# Supplementary Figure 1

A

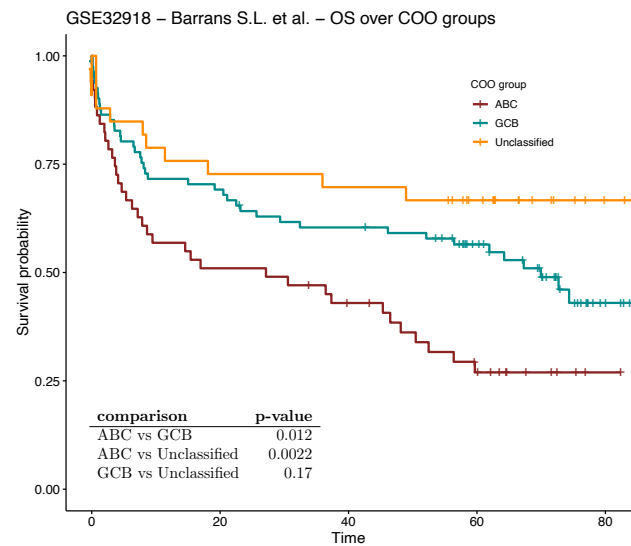

B

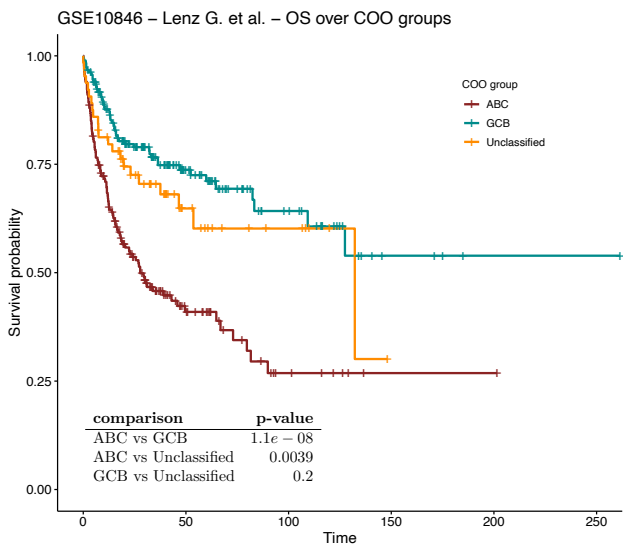

C

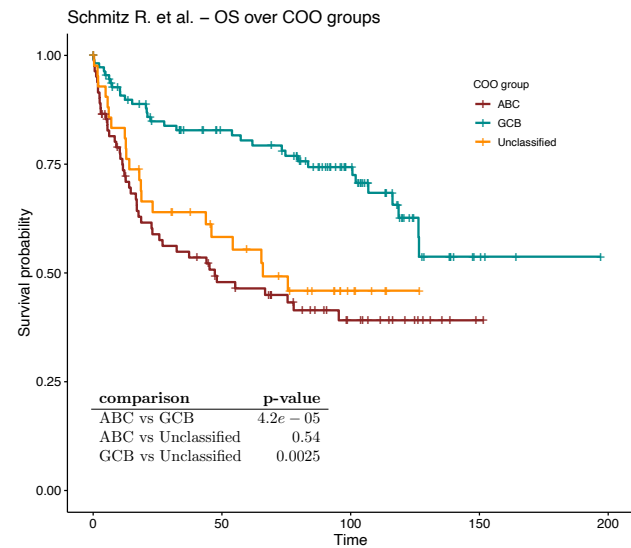

D

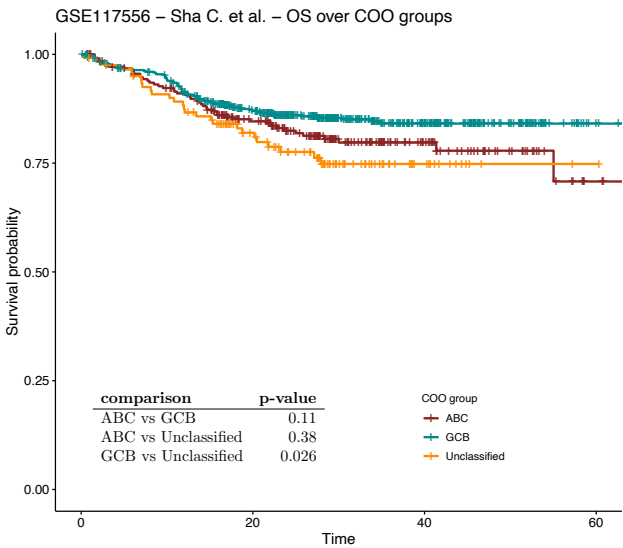

E

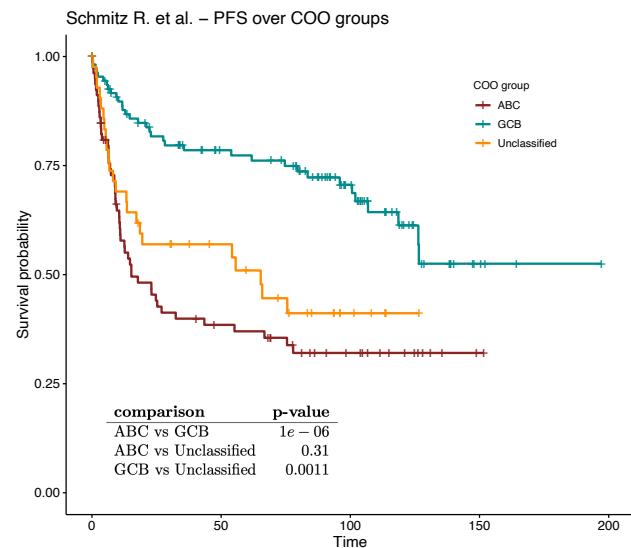

F

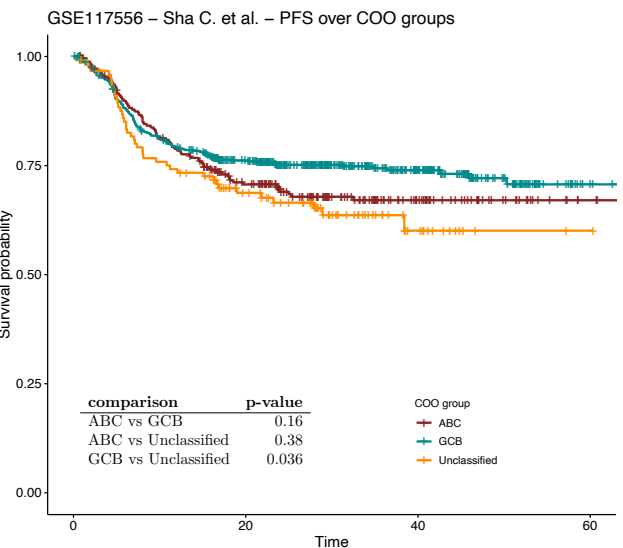

Supplement: Supplementary file 1 [file Image_1.pdf]
